# Supplementary material for: Sepsis—A Retrospective Cohort Study of Bloodstream Infections
Source: Antibiotics (Basel). 2020 Nov 28;9(12):851. doi: 10.3390/antibiotics9120851 (PMC7760988; doi:10.3390/antibiotics9120851)
Supplement: Supplementary file 1 [file antibiotics-09-00851-s001.pdf]

YEAR	2015		2016		2017		2018		2019	
**ISOLATED SPECIES**	**n. patients**	**percentage value**	**n. patients**	**percentage value**	**n. patients**	**percentage value**	**n. patients**	**percentage value**	**n. patients**	**percentage value**
Staphylococcus epidermidis	114	16.38%	139	18.94%	150	17.88%	133	15.32%	149	18.35%
Escherichia coli	73	10.49%	74	10.08%	110	13.11%	87	10.02%	87	10.71%
Klebsiella pneumoniae	65	9.34%	65	8.86%	63	7.51%	91	10.48%	27	3.33%
Staphylococcus aureus	57	8.19%	103	14.03%	91	10.85%	158	18.20%	103	12.68%
Staphylococcus hominis	53	7.61%	57	7.77%	68	8.10%	52	5.99%	45	5.54%
Enterococcus faecalis	48	6.90%	37	5.04%	44	5.24%	49	5.65%	44	5.42%
Staphylococcus haemolyticus	46	6.61%	66	8.99%	65	7.75%	49	5.65%	53	6.53%
Acinetobacter baumannii	39	5.60%	31	4.22%	49	5.84%	54	6.22%	29	3.57%
Proteus mirabilis	24	3.45%	10	1.36%	20	2.38%	7	0.81%	14	1.72%
Pseudomonas aeruginosa	24	3.45%	29	3.95%	10	1.19%	23	2.65%	34	4.19%
Candida albicans	19	2.73%	9	1.23%	14	1.67%	13	1.50%	17	2.09%
Enterococcus faecium	15	2.16%	12	1.63%	17	2.03%	25	2.88%	14	1.72%
Enterobacter Cloacae	14	2.01%	9	1.23%	8	0.95%	14	1.61%	9	1.11%
Candida parapsilosis	13	1.87%	8	1.09%	7	0.83%	0	0.00%	11	1.35%
Staphylococcus capitis	11	1.58%	14	1.91%	11	1.31%	5	0.58%	19	2.34%
Staphylococcus warneri	8	1.15%	6	0.82%	6	0.72%	3	0.35%	4	0.49%
Candida glabrata	6	0.86%	0	0.00%	4	0.48%	2	0.23%	1	0.12%
Serratia marcescens	5	0.72%	5	0.68%	7	0.83%	8	0.92%	6	0.74%
Staphylococcus simulans	5	0.72%	4	0.54%	4	0.48%	11	1.27%	3	0.37%
Enterobacter aerogenes	4	0.57%	0	0.00%	1	0.12%	3	0.35%	0	0.00%
Providencia stuartii	4	0.57%	3	0.41%	14	1.67%	9	1.04%	1	0.12%
Staphylococcus auricularis	4	0.57%	2	0.27%	2	0.24%	1	0.12%	0	0.00%
Candida tropicalis	3	0.43%	1	0.14%	3	0.36%	0	0.00%	5	0.62%
Enterococcus avium	3	0.43%	1	0.14%	0	0.00%	0	0.00%	1	0.12%
Stenotrophomonas maltophilia	3	0.43%	3	0.41%	7	0.83%	3	0.35%	2	0.25%
Streptococcus gordonii	3	0.43%	1	0.14%	2	0.24%	0	0.00%	1	0.12%
Streptococcus mitis	3	0.43%	5	0.68%	2	0.24%	2	0.23%	4	0.49%
Streptococcus pneumoniae	3	0.43%	1	0.14%	2	0.24%	5	0.58%	0	0.00%
Citrobacter koseri	2	0.29%	0	0.00%	1	0.12%	8	0.92%	12	1.48%
Klebsiella oxytoca	2	0.29%	1	0.14%	3	0.36%	2	0.23%	9	1.11%
Morganella morganii	2	0.29%	4	0.54%	1	0.12%	1	0.12%	3	0.37%
Pseudomonas fluorescens	2	0.29%	3	0.41%	0	0.00%	0	0.00%	0	0.00%
Salmonella ser.Typhi	2	0.29%	0	0.00%	0	0.00%	0	0.00%	0	0.00%
Staphylococcus lugdunensis	2	0.29%	0	0.00%	4	0.48%	1	0.12%	2	0.25%
Streptococcus gallolyticus ssp pasteurianus	2	0.29%	1	0.14%	0	0.00%	2	0.23%	0	0.00%
Achromobacter denitrificans	1	0.14%	1	0.14%	2	0.24%	0	0.00%	0	0.00%
Aeromonas hydrophila	1	0.14%	1	0.14%	1	0.12%	0	0.00%	0	0.00%
Alcaligenes faecalis	1	0.14%	0	0.00%	0	0.00%	0	0.00%	0	0.00%
Enterococcus durans	1	0.14%	0	0.00%	1	0.12%	1	0.12%	1	0.12%
Enterococcus gallinarum	1	0.14%	0	0.00%	2	0.24%	0	0.00%	1	0.12%
Leclercia adecarboxylata	1	0.14%	0	0.00%	0	0.00%	0	0.00%	0	0.00%
Staphylococcus cohnii ssp cohnii	1	0.14%	0	0.00%	0	0.00%	0	0.00%	0	0.00%
Staphylococcus cohnii ssp urealyticus	1	0.14%	0	0.00%	0	0.00%	0	0.00%	1	0.12%
Staphylococcus lentus	1	0.14%	0	0.00%	2	0.24%	0	0.00%	1	0.12%
Staphylococcus saprophyticus	1	0.14%	2	0.27%	1	0.12%	1	0.12%	2	0.25%
Streptococcus agalactiae	1	0.14%	2	0.27%	3	0.36%	0	0.00%	2	0.25%
Streptococcus gallolyticus	1	0.14%	1	0.14%	3	0.36%	13	1.50%	4	0.49%
Streptococcus sanguinis	1	0.14%	4	0.54%	2	0.24%	1	0.12%	4	0.49%
Achromobacter xylosoxidans	0	0.00%	0	0.00%	2	0.24%	0	0.00%	0	0.00%
Acinetobacter lwoffii	0	0.00%	1	0.14%	0	0.00%	0	0.00%	0	0.00%
Aeromonas sobria	0	0.00%	0	0.00%	1	0.12%	0	0.00%	0	0.00%
Burkholderia cepacia	0	0.00%	1	0.14%	0	0.00%	0	0.00%	0	0.00%
Candida guilliermondii	0	0.00%	0	0.00%	1	0.12%	14	1.61%	0	0.00%
Candida norvegensis	0	0.00%	0	0.00%	1	0.12%	5	0.58%	0	0.00%
Citrobacter freundii	0	0.00%	1	0.14%	0	0.00%	1	0.12%	1	0.12%
Enterobacter cloacae	0	0.00%	0	0.00%	5	0.60%	0	0.00%	7	0.86%
Enterococcus casseliflavus	0	0.00%	0	0.00%	0	0.00%	0	0.00%	1	0.12%
Enterococcus hirae	0	0.00%	0	0.00%	1	0.12%	0	0.00%	0	0.00%
Klebsiella ssp ozaenae	0	0.00%	0	0.00%	2	0.24%	0	0.00%	67	8.25%
Pantoea agglomerans	0	0.00%	0	0.00%	2	0.24%	0	0.00%	0	0.00%
Pseudomonas putida	0	0.00%	1	0.14%	0	0.00%	0	0.00%	1	0.12%
Salmonella spp	0	0.00%	3	0.41%	3	0.36%	2	0.23%	2	0.25%
Serratia ficaria	0	0.00%	1	0.14%	2	0.24%	0	0.00%	0	0.00%
Serratia liquefaciens	0	0.00%	1	0.14%	2	0.24%	0	0.00%	2	0.25%
Sphingomonas paucimobilis	0	0.00%	1	0.14%	0	0.00%	0	0.00%	0	0.00%
Staphylococcus gallinarum	0	0.00%	3	0.41%	0	0.00%	0	0.00%	0	0.00%
Staphylococcus hyicus	0	0.00%	0	0.00%	1	0.12%	0	0.00%	0	0.00%
Staphylococcus intermedius	0	0.00%	0	0.00%	1	0.12%	0	0.00%	1	0.12%
Staphylococcus schleiferi	0	0.00%	1	0.14%	0	0.00%	0	0.00%	1	0.12%
Staphylococcus xylosus	0	0.00%	0	0.00%	0	0.00%	1	0.12%	1	0.12%
Streptococcus anginosus	0	0.00%	2	0.27%	1	0.12%	0	0.00%	0	0.00%
Streptococcus dysgalactiae ssp equisimilis	0	0.00%	2	0.27%	0	0.00%	0	0.00%	0	0.00%
Streptococcus infantarius	0	0.00%	0	0.00%	1	0.12%	0	0.00%	0	0.00%
Streptococcus mutans	0	0.00%	0	0.00%	0	0.00%	2	0.23%	1	0.12%
Streptococcus parasanguinis	0	0.00%	1	0.14%	4	0.48%	1	0.12%	1	0.12%
Streptococcus pyogenes	0	0.00%	0	0.00%	1	0.12%	2	0.23%	1	0.12%
Streptococcus salivarius	0	0.00%	0	0.00%	1	0.12%	1	0.12%	0	0.00%
Yersinia frederiksenii	0	0.00%	0	0.00%	0	0.00%	2	0.23%	0	0.00%
